# PM_2.5_ Exposure and Intrauterine Inflammation: A Possible Mechanism for Preterm and Underweight Birth

**DOI:** 10.1289/ehp.124-A190

**Published:** 2016-10-01

**Authors:** Nate Seltenrich

**Affiliations:** Nate Seltenrich covers science and the environment from Petaluma, CA. His work has appeared in *High Country News*, *Sierra*, *Yale Environment 360*, *Earth Island Journal*, and other regional and national publications.

Intrauterine inflammation (IUI) is a risk factor for a variety of adverse birth outcomes,^1^ ^,^ ^2^ ^,^ ^3^ and some investigators have hypothesized it could also play a role in the risk of being born preterm or underweight.^4^ ^,^ ^5^ ^,^ ^6^ Several other studies have demonstrated that a pregnant woman’s exposure to fine particulate matter (PM_2.5_) appears to increase her baby’s risk of being born preterm or underweight.^7^ ^,^ ^8^ ^,^ ^9^ A new study bridges these lines of inquiry and offers evidence that IUI is associated with exposure to PM_2.5_.^10^ Coauthor Marsha Wills-Karp, a professor of environmental health sciences at The Johns Hopkins University, says, “The study gives us some indication that there’s an actual change in the placenta and … inflammation occurring in close proximity to the fetus that is associated with exposure to air pollution.”

**Figure:**
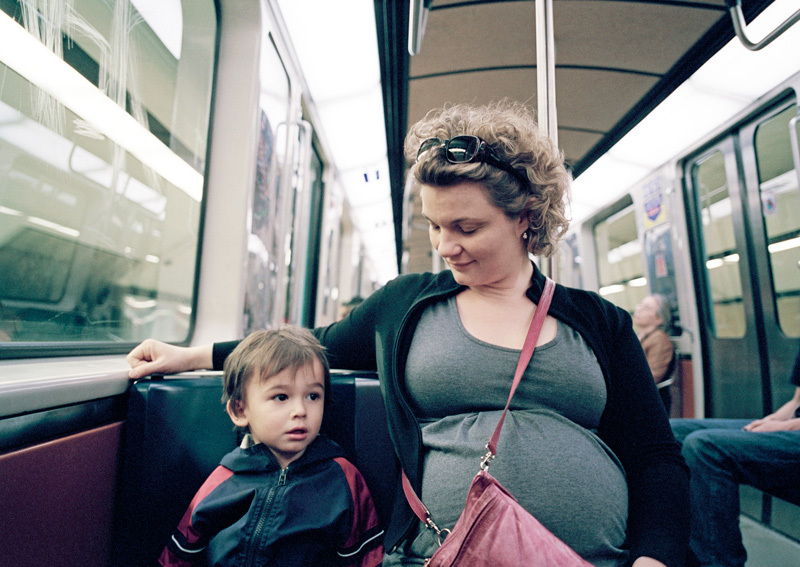
When researchers estimated pregnant women’s exposures to PM2.5, they found that higher exposures were associated with increased incidence of intrauterine inflammation. Although the association was seen with exposures in all trimesters, it was weaker among women exposed during the second and third trimesters. © Roderick Chen/Getty Images

IUI is a condition in which some area of the placenta becomes inflamed independent of any clinical infection. It is estimated to occur in 25–50% of preterm births and 20% of full-term births,^11^ although data are sparse, as sensitive detection methods are not routinely used.^10^ Maternal fever at birth is a clue that IUI may be present,^12^ but it can also indicate other conditions, and it does not occur in all cases of IUI.^13^

The researchers analyzed more than 5,000 mother–child pairs who were part of the larger Boston Birth Cohort. The births occurred at Boston Medical Center between 1999 and 2012. To estimate the women’s daily PM_2.5_ exposures during and just prior to pregnancy, the team used historical data from 13 air quality monitors located throughout Massachusetts. And to assess IUI, they relied on both fever during labor and placental pathology.

The results showed a positive association between PM_2.5_ exposure and incidence of IUI for each trimester. Exposures estimated to have occurred during the first trimester were the most strongly associated with IUI at birth. The women with the highest exposures during this period were nearly twice as likely as the least-exposed women to exhibit IUI months later at birth—even though fewer than one-third of the women are thought to have ever seen PM_2.5_ levels exceeding the federal annual standard.^14^ Exposure during the three months before conception was also associated with IUI at birth, although the association was no longer evident after the researchers adjusted for exposure during other time periods.^10^

Beate Ritz, a researcher and professor of epidemiology at the University of California, Los Angeles, says the new findings suggest causation in the somewhat murky relationship between PM_2.5_ and low birth weight and preterm birth. “I think what we really need is more intermediate biomarkers, and that’s what this group did. These are the kinds of biological links that we need in the field to really get this confirmed,” says Ritz, who was not associated with the study. “This is a step forward toward a biological pathway, and inflammation is certainly a very logical one.”

The researchers could not tell whether IUI observed at birth had originated early in the pregnancy, within the last week, or somewhere in between. So it is still too soon to consider IUI a sensitive biomarker for assessing early biological effects of PM_2.5_. Nonetheless, the results may be useful for identifying times in pregnancy for more intensive follow-up in a future clinical study.

Mariana Matera Veras, an experimental researcher in air pollution at the University of São Paolo, who also was not involved with the study, agrees that further studies are needed to evaluate the progression of IUI during pregnancy. She praises the authors for taking pregestational exposures into account and reiterates that IUI was seen in association with exposures that were below the accepted limits. “I think ‘safe’ levels of air pollution do not exist,” she says.

The study also calls attention to the placenta as a potential source of epidemiological data, says coauthor Xiaobin Wang, director of the Center on Early Life Origins of Disease at the Johns Hopkins University Bloomberg School of Public Health. “The placenta is such a vital organ,” she says, yet researchers traditionally have not had access to it. “This often-discarded organ may have some real value in assessing early health effect of environmental exposure.”

Granted, in this case there’s a limit to what placental pathology can reveal about PM_2.5_ intake and IUI onset: “It doesn’t completely tell us how this is occurring,” notes Wills-Karp. “To do more mechanistic studies, we’d have to obtain live tissue by culturing placenta cells rather than simply examining pathology slides.”
